# A Case Report of an Elderly Patient With Cushing’s Disease and Multiple Chronic Conditions

**DOI:** 10.7759/cureus.63277

**Published:** 2024-06-27

**Authors:** Ning Zhang, Yuchen Wei, Xuan Qu, Lin Kang, Xiaohong Liu

**Affiliations:** 1 Department of Geriatrics, Peking Union Medical College Hospital, Peking Union Medical College, Chinese Academy of Medical Sciences, Beijing, CHN; 2 Department of Endocrinology, Peking Union Medical College Hospital, Peking Union Medical College, Chinese Academy of Medical Sciences, Beijing, CHN

**Keywords:** chronic obstructive pulmonary disease (copd), treatment choices, multimorbidity, older adult, cushing’s disease

## Abstract

Cushing's disease (CD) is a rare and serious condition characterized by a persistent increase in cortisol levels, resulting in various complications across multiple bodily systems. Elderly individuals often face a multitude of chronic illnesses and geriatric syndromes, which can complicate the diagnosis and treatment of CD in this demographic. This case study details the presentation of an elderly patient with adrenocorticotropic hormone (ACTH)-dependent CD, who initially presented with an acute exacerbation of chronic obstructive pulmonary disease. The article delves into the unique onset characteristics and treatment strategies for CD in the elderly, providing valuable insights for the comprehensive management of similar clinical cases.

## Introduction

Cushing’s disease (CD) is characterized by a corticotroph pituitary adenoma that overproduces adrenocorticotrophic hormone (ACTH). Clinical manifestations of CD include weight gain, central fat distribution, skin striae, thinning skin, muscle loss, and fatigue. Comorbidities associated with hypercortisolism, such as diabetes mellitus, hypertension, cardiovascular disease, and deep venous thrombosis, can lead to severe complications and increased mortality rates [1]. CD predominantly affects young women and is less frequently seen in elderly patients. Elderly patients commonly present with a combination of multiple chronic diseases and geriatric syndromes. The coexistence of CD in the elderly further complicates diagnosis and management due to the intricate nature of these conditions [2]. This article presents a case study of an elderly individual with CD alongside multiple chronic diseases and geriatric syndromes.

## Case presentation

**Figure 1:**
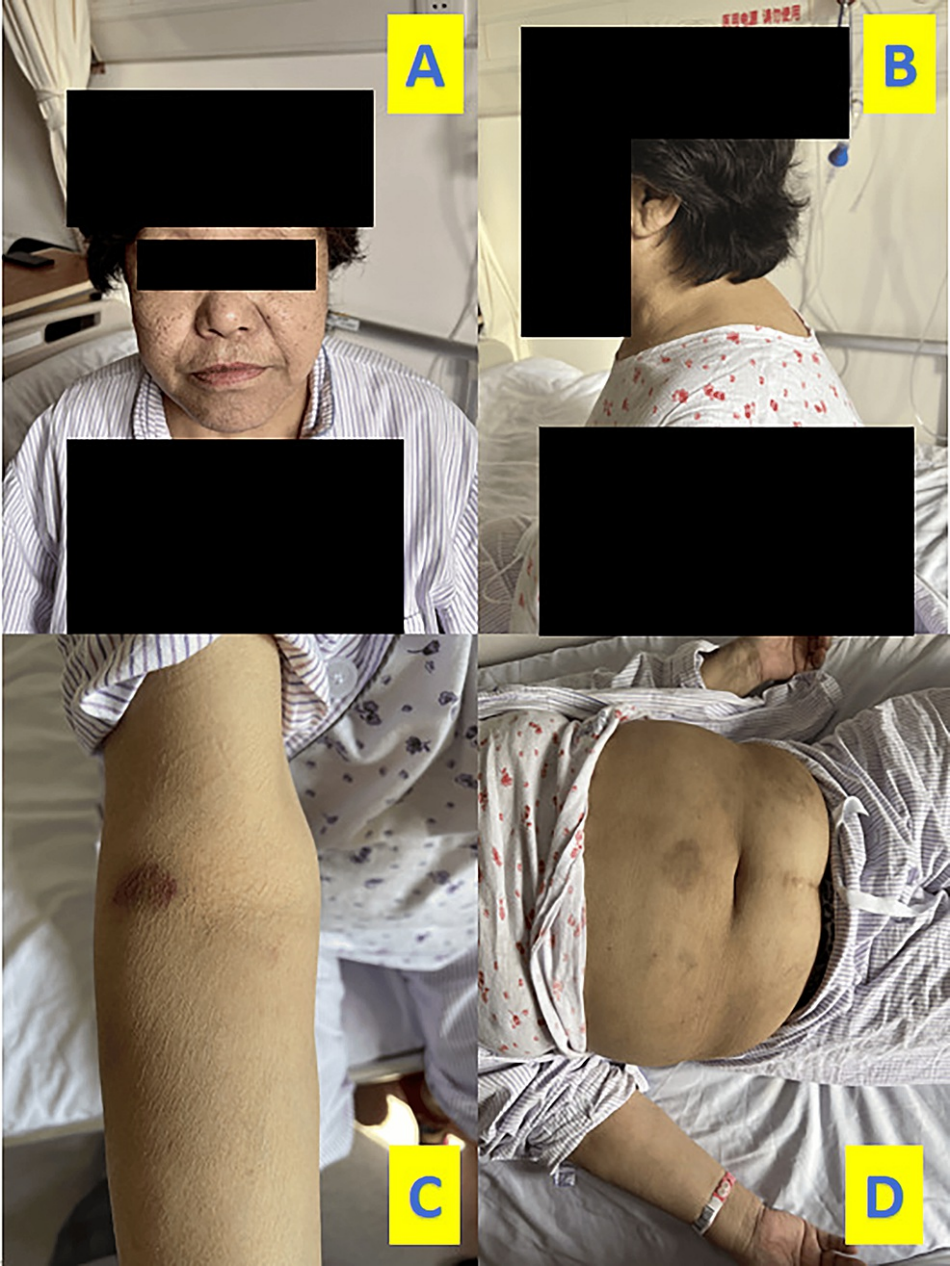
(A-D) Physical signs observed in the patient upon admission. Figure 1A reveals no signs of a full moon face, while Figure 1B shows a buffalo back. Figure 1C displays thin skin with visible ecchymosis, and Figure 1D exhibits abdominal fat accumulation without purple streaks on the skin.

A 72-year-old female patient was admitted to the geriatric medicine ward of Peking Union Medical College Hospital due to a 20-year history of intermittent coughing and wheezing that recently worsened, accompanied by general edema for eight days. Since late autumn and winter two decades ago, the patient has been experiencing shortness of breath, coughing, sputum production, and wheezing on and off every year. Initially diagnosed with chronic bronchitis at a local hospital, the patient typically received intravenous cefuroxime for seven to 10 days when symptoms flared, leading to relief. About nine days prior to admission, during the winter season, the patient developed shortness of breath after catching a cold while shopping, along with coughing and yellow-white sputum, and a noticeable decrease in activity tolerance. Seeking treatment at a local hospital two days after the above-mentioned symptoms, tests revealed elevated white blood cell count and chest CT findings suggestive of pulmonary infection. Despite initial treatment with ceftriaxone sodium and budesonide inhalation, symptoms persisted, limiting mobility to bed rest and necessitating sitting upright at night to breathe. General edema developed gradually, along with weakness in both lower limbs. Notably, there was no history of corticosteroid use. Potassium levels were found to be low (2.48 mmol/L) but improved with oral supplementation, leading to alleviation of lower limb weakness. Past medical history indicated a 20-year history of hypertension with no history of diabetes. The patient has resided in the cold suburbs of Hulunbuir, Inner Mongolia, where winter temperatures average -30°C. She smoked 10-20 cigarettes per day for 30 years before quitting and has had significant exposure to charcoal fire and soot. Upon admission, physical examination revealed a pulse rate of 92 times/min, blood pressure of 140/87 mmHg, oxygen saturation of 89% on room air, BMI of 33.9 kg/m^2^, abdominal obesity, and other physical findings such as supraclavicular fat pad, buffalo back, thin skin, pigmentation in the armpits, and scattered ecchymoses on the upper limbs (Figure 1). The patient exhibited normal visual acuity and visual field, no sternum or rib tenderness, an enlarged tongue without tremor, a barrel-shaped chest, symmetrical respiratory movements in both lungs with biphasic wheezing mainly during expiration, and level V muscle strength in both lower limbs. A comprehensive geriatric assessment revealed 4 points for activities of daily living (ADL), 3 points for instrumental activities of daily living (IADL), 2 points on the NRS-2002 scale, dominant hand grip strength of 15.1 kg, and a Fried score of 3 points indicating frailty.

**Figure 2:**
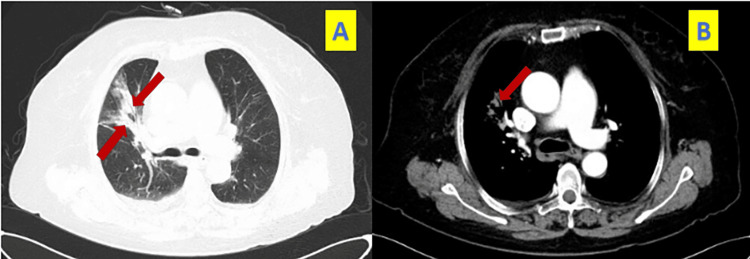
The patient's enhanced chest CT revealed an enlargement in the front-to-back diameter of the thorax, along with various patches and consolidation in the middle lobe of the right lung. Additionally, a positive air bronchogram was observed, highlighted by the red arrow in images A and B corresponding to the CT lung and mediastinal windows, respectively.

**Figure 3:**
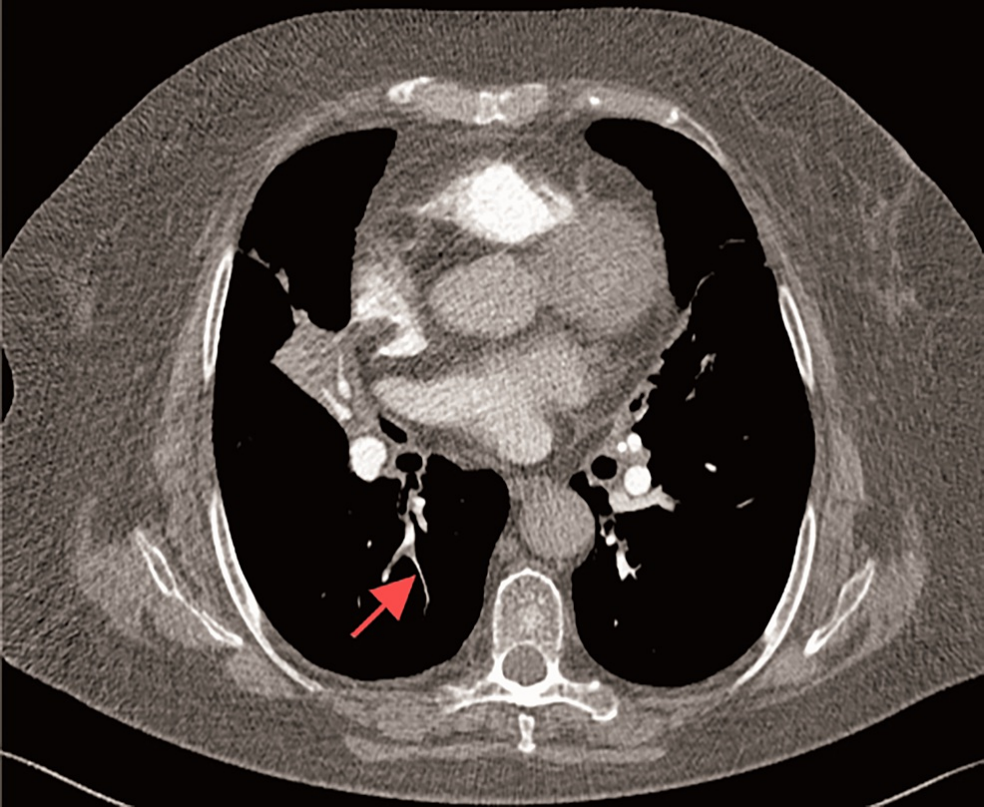
The patient's computed tomography pulmonary angiography (CTPA) revealed a low-density filling defect in the pulmonary artery within the dorsal segment of the right lower lobe of the lung, indicative of chronic pulmonary embolism (red arrow).

Following admission, additional examinations were performed. Blood work showed platelets at 176×10^9^/L, white blood cells at 7.70×10^9^/L, neutrophils at 77.3%, and hemoglobin at 113 g/L. High-sensitivity C-reactive protein (hsCRP) levels were elevated at 6.69 mg/L. The lipid profile indicated total cholesterol at 4.72 mmol/L, total triglycerides at 1.81 mmol/L, and low-density lipoprotein cholesterol (LDL-C) at 2.49 mmol/L. Glycated hemoglobin (HbA1c) was elevated at 7.8%. D-dimer levels were increased at 2.15 mg/L FEU. Lymphocyte subset analysis revealed a decrease in CD4+T cell percentage (29.7%), CD4+T cell count (249/μL), and CD8+T cell count (194/μL). Deep vein ultrasound of both lower limbs showed no evidence of thrombosis. An enhanced CT scan of the chest showed an increase in the anteroposterior diameter of the thorax, the presence of multiple patches and consolidation shadows in the right lung's middle lobe, multiple patches and cord shadows in the lower lobes of both lungs, and bronchial stenoses (Figure 2). A contrast-enhanced scan revealed a slightly reduced distal pulmonary vascular shadow in the right lower lobe and a widening of the main pulmonary artery trunk. CT pulmonary angiography identified a low-density filling defect in the pulmonary artery of the dorsal segment of the right lower lobe, suggestive of chronic pulmonary embolism (Figure 3). Pulmonary function tests demonstrated obstructive ventilatory dysfunction (FEV1/FVC=55%), reduced diffusing function, and a negative diastolic test.

**Figure 4:**
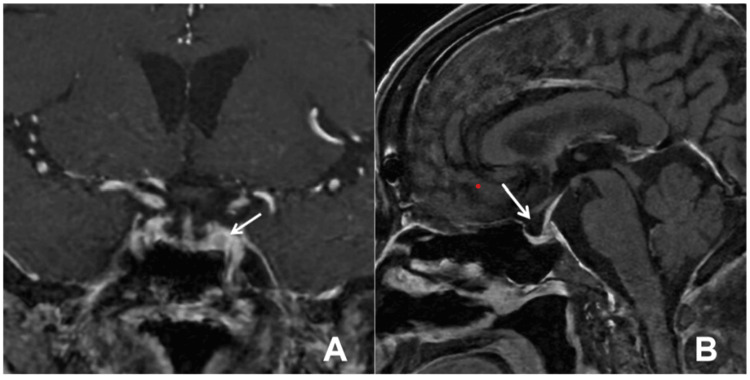
High-resolution MRI images of the patient's pituitary gland. 4A depicts a round-like T1-phase low-signal adenoma located on the left side of the pituitary gland, while 4B shows a sagittal view of the vacuolated sella turcica.

Endocrine-related examinations were performed, showing a morning blood cortisol level of 17.1 μg/dL, elevated blood ACTH at 93.9 pg/mL, and a 24-hour urine-free cortisol (24-hour UFC) level of 3,485.0 μg/24 hours (1.5 mg of dexamethasone was orally administered on the day of urine collection). Subsequent tests indicated an increase in total blood cortisol levels at 65.5 μg/dL (8 AM) and 51.800 μg/dL (0 AM), elevated blood ACTH at 192.0 pg/mL, and 24-hour UFC at 3,579.8 μg/24 hours. Imaging studies, including octreotide imaging, did not reveal any significant abnormalities. A plain scan of the pituitary gland + dynamic enhanced MRI revealed a thin pituitary gland with partial vacuolation of the sella turcica. Octreotide imaging did not show any obvious abnormalities. The inferior petrosal sinus sampling (IPSS) + desmopressin (DDAVP) stimulation test indicated a baseline left central/peripheral ACTH ratio of 1.47, which increased to 6.43 after DDAVP stimulation (Table 1). High-resolution MRI of the pituitary gland identified a round T1-phase low-intensity adenoma on the left side (Figure 4). In the context of bone metabolism, the patient exhibits low levels of total 25-hydroxyvitamin D at 6.0 ng/mL, elevated levels of beta-isomerized C-terminal telopeptide (β-CTX) at 1.15 ng/mL, increased parathyroid hormone (PTH) at 98.9 pg/mL, and total procollagen type 1 amino-terminal propeptide (tP1NP) at 16.5 ng/mL. The bone density measurements (T value) indicate -0.7 for the lumbar spine L1-L4 and -1.7 for the femur. An evaluation of anterior pituitary function reveals thyroid function with thyroid-stimulating hormone (TSH) at 1.294 μIU/mL, free thyroxine (FT4) at 1.00 ng/dL, and free triiodothyronine (FT3) at 2.26 pg/mL. Furthermore, sex hormone levels show follicle-stimulating hormone (FSH) at 1.56 IU/L, below the menopausal reference value of > 40 IU/L, luteinizing hormone (LH) below 0.2 IU/L (normal range: 10.87-58.64 IU/L), prolactin (PRL) at 7.1 ng/mL (normal range: < 30 ng/mL), progesterone at 1.24 ng/mL above the normal range of < 0.78 ng/mL, E2 at 36 pg/mL above the normal range of < 25 pg/mL, T at 0.68 ng/mL above the normal range of 0.10-0.66 ng/mL, and insulin-like growth factor IGF-1 at 65 ng/mL.

**Table 1:** Patient's inferior petrosal sinus sampling (IPSS) + desmopressin (DDAVP) experimental results (unit: ng/L).

ACTH	Peripheral sampling	Left inferior petrosal sinus sampling	Left inferior petrosal sinus sampling/peripheral	Right inferior petrosal sinus sampling	Right inferior petrosal sinus sampling/peripheral
0 min	109.0	160.0	1.47	132.0	1.21
3 min	83.7	538.0	6.43	263.0	3.14
5 min	181.0	479.0	2.65	301.0	1.66
10 min	235.0	340.0	1.45	295.0	1.26

The patient was diagnosed with acute exacerbation of chronic obstructive pulmonary disease (COPD) secondary to pulmonary infection (AE-COPD) based on chest imaging, lung function findings, and a history of heavy smoking. The treatment plan included inhalation of budesonide, oral acetylcysteine with myrtle oil to eliminate phlegm, and consultation with the rehabilitation department for active circulation breathing and sputum excretion training. Antibiotics such as cefaclor and amoxicillin were administered for lung infection, along with oral ciprofloxacin for a two-week course. As a result of the treatment, the patient's dyspnea symptoms improved, and cough and sputum production decreased. Diuretic therapy with furosemide and spironolactone, in addition to low-flow oxygen therapy, was initiated to address edema in both lower limbs and generalized edema suspected to be due to right heart failure secondary to COPD. The patient's weight decreased from 85 kg to 80 kg, and the edema subsided. In the context of CD, the primary treatment option typically recommended is surgical intervention involving pituitary tumor resection using the nasosphenoidal approach. However, in this particular case, the patient's compromised cardiopulmonary function and pulmonary infection presented challenges in tolerating general anesthesia. Additionally, the patient's pituitary MRI revealed a vacuolated sella turcica, increasing the risk of cerebrospinal fluid leakage during surgery. Following a thorough discussion between the medical team and the patient, it was mutually agreed to postpone surgical intervention. Instead, the patient was started on mifepristone therapy, beginning with a low dose of 25 mg tid and gradually escalating to 50 mg tid, to counteract the effects of glucocorticoids. Remarkably, the patient did not experience any gastrointestinal adverse effects, such as nausea, vomiting, or anorexia. Imaging studies revealed a low-density filling defect in the pulmonary artery of the right lower lobe, which was suggestive of chronic pulmonary embolism. Subsequently, anticoagulant therapy with rivaroxaban 15 mg/d was initiated. Additionally, the patient experienced a notable decrease in CD4+ T cell count, indicating immunosuppression. To prevent opportunistic infections, cotrimoxazole 1#/d was added to the treatment regimen. Following a month of comprehensive management, the patient's condition significantly improved, leading to a successful discharge from the hospital.

## Discussion

Cushing's syndrome (CS) refers to a group of disorders characterized by the overproduction of glucocorticoids, particularly cortisol, from the adrenal glands. The primary cause is often the excessive secretion of ACTH by the pituitary gland, leading to increased cortisol production in the adrenal cortex. This specific form of CS is known as CD and is observed in approximately 70% of all CS cases [3].

The patient initially presented with an acute exacerbation of COPD due to a pulmonary infection, which was followed by the development of weakness in both lower limbs. Subsequent blood tests revealed hypokalemia and elevated cortisol levels, prompting consideration of CS. Diagnosis of CS typically involves assessing clinical symptoms and conducting specific laboratory tests, such as midnight salivary cortisol levels, 24-hour urinary-free cortisol (UFC) levels, and overnight low-dose dexamethasone suppression tests [4]. Following admission, the patient's 24-hour urinary-free cortisol levels notably increased. Notably, the patient had taken 1.5 mg of dexamethasone for an enhanced CT scan on the day of the initial urinary-free cortisol test. Despite this, cortisol secretion remained unimpeded, indicating autonomous cortisol production characteristic of CS.

CS can be categorized into ACTH-dependent and ACTH-independent forms based on ACTH levels. In ACTH-dependent CS, ACTH levels are typically > 20 pg/mL, while, in ACTH-independent CS, ACTH levels are usually < 10 pg/mL. In this case, the patient's ACTH level at 8 AM was 54.1 pg/mL, indicating a diagnosis of ACTH-dependent CS. The primary cause of ACTH-dependent cases is CD, followed by ectopic ACTH syndrome (EAS). Ectopic secretion tumors in EAS are most commonly found in the lungs, such as small cell lung cancer or lung carcinoid tumors. Despite no suspicious lesions being identified on contrast-enhanced chest CT upon admission, the initial consideration was CD. Subsequent pituitary imaging revealed a thin pituitary gland, a vacuated sella turcica, and no apparent tumor lesions. Bilateral inferior petrosal sinus sampling (BIPSS) was then conducted on the patient. The inferior petrosal sinus vein functions as the drainage vein for the pituitary gland, allowing the release of hormones. In individuals with CD, the concentration of ACTH in the inferior petrosal sinus vein is significantly higher compared to peripheral veins. This disparity is used to calculate central and peripheral ACTH levels, termed inferior petrosal sinus sampling/peripheral (IPS/P) ACTH. The ratio of these concentrations assists in localizing and diagnosing CS. DDAVP prompts ACTH secretion by binding to receptors on pituitary tumors. During the BIPSS experiment, DDAVP stimulation can improve diagnostic sensitivity and specificity. The current international recommendation is to utilize an ACTH IPS/P ratio cutoff of ≥ 2 (pre-provocation) and IPS/P ≥ 3 (post-DDAVP provocation) for diagnosing CD. However, if clinical suspicion persists despite negative results at these thresholds, the Endocrinology Department of Peking Union Medical College Hospital proposed new diagnostic criteria in 2023: IPS/P ≥ 1.4 before stimulation and IPS/P ≥ 2.8 after stimulation, showing high sensitivity and specificity in diagnosing CD [5]. This patient meets the new diagnostic criteria, confirming the diagnosis of CD. Subsequent high-resolution MRI of the pituitary gland revealed a left-sided pituitary adenoma, further confirming the initial suspicion.

This patient had a history of using glucocorticoid inhalation therapy during the course of the disease, which needed to be distinguished from exogenous CS. The patient had only used inhaled glucocorticoids for two days prior to admission. Following admission, the patient was unable to discontinue the use of inhaled glucocorticoid-containing medication due to her condition, resulting in a lack of data on blood cortisol levels without exogenous glucocorticoids being collected during the course of the disease. However, since short-term use of inhaled glucocorticoids typically does not lead to a significant increase in cortisol levels as seen in this patient [6], and blood ACTH levels should be suppressed due to negative feedback, which contradicts the patient's test results, it suggests that the patient is experiencing autonomous cortisol secretion within her body.

The clinical manifestations of CS primarily involve the following:

(1) Glucolipid metabolism +disorder: Cortisol inhibits glucose uptake and utilization by peripheral tissues, promotes gluconeogenesis, raises blood sugar levels, induces insulin resistance (IR), interferes with lipid metabolism, and enhances fat synthesis.

(2) Water and salt metabolism disorder: Cortisol retains sodium, eliminates potassium, and mimicks mineralocorticoid effects, resulting in water and sodium retention while increasing urinary potassium excretion, leading to elevated blood pressure and reduced blood potassium levels.

(3) Abnormal bone metabolism: Excessive cortisol levels boost urinary calcium excretion and stimulate osteoclast activity, causing bone demineralization, osteoporosis, and heightened fracture risk.

(4) Opportunistic infections: Hypercortisolemia suppresses the immune system, making individuals susceptible to opportunistic infections.

(5) Coagulation events: Patients with CS exhibit a hypercoagulable state, leading to an 18-fold higher incidence of venous thromboembolism (VTE) compared to the general population [7]. This increased risk of VTE is linked to elevated levels of coagulation factors VIII and IX, von Willebrand factor, fibrinogen, and plasminogen activator inhibitor-1 [8]. Post-admission lung imaging of the patient revealed chronic pulmonary embolism, believed to be connected to the hypercoagulable state secondary to CD.

(6) Impact on other anterior lobe functions of the pituitary gland: Pituitary tumors can lead to compression of normal pituitary tissue, potentially causing hypofunction of the anterior lobe. Although the patient's TSH and IGF-1 levels were within normal range, lower LH and FSH levels indicate possible compression by the pituitary gland tumor. It is important to also take into account the inhibitory impact of elevated cortisol levels on the gonadal hormone axis. Therefore, regular monitoring is essential to reassess abnormal hormone levels in the patient.

The prevalence of CS in the elderly is relatively low, with limited research available on the topic. A multicenter study conducted by Amodru et al. [9] revealed that only a small proportion of elderly patients (≥ 65 years old) were diagnosed with CS, accounting for 9.8% of the total 1972 patients included in the study. Clinical presentations in the elderly lack the typical signs of cortisol excess but show an increase in coexisting conditions and muscle weakness [9]. Another study by Damanti et al. found that, among 545 patients with CD, only 8.3% were elderly (≥ 60 years old) [10]. Most cases of CS in the elderly are often discovered incidentally. As individuals age, there is an increase in body fat levels, muscle fibers undergo atrophy, and muscle mass decreases. The bioavailability of calcium decreases in the elderly, along with insufficient synthesis of parathyroid hormone and vitamin D, which regulate calcium metabolism, making the body more vulnerable to deficiencies. This reduction in calcium and total bone minerals can lead to weakened and brittle bones, increasing the risk of fractures. Moreover, there is an increase in the activity of fat-synthesizing enzymes and a decrease in decomposing enzymes, resulting in greater fat accumulation. Additionally, the aging process leads to a slower rate of amino acid conversion and decreased protein anabolism. Natural skin aging in the elderly is characterized by thinning, dryness, fine wrinkles, reduced elasticity, and abnormal pigmentation [11]. These age-related changes make it challenging to differentiate the clinical features of CS from normal aging [8].

Transsphenoidal pituitary tumor resection is typically considered the primary treatment option for individuals with pituitary CS. The postoperative remission rate is reported to be around 75%, with a low recurrence rate of 3.2% after 18 months [12]. However, in cases where the pituitary MRI reveals a vacuolated sella turcica, there may be challenges in identifying an underlying pituitary tumor. While surgery can lead to a cure in 70%-90% of cases, patients with CD and vacuolated sella turcica are at a higher risk of postoperative complications such as diabetes insipidus, hypopituitarism, and cerebrospinal fluid leakage [13]. For individuals who are not suitable candidates for surgical resection, drug therapy, particularly the use of mifepristone, can be considered. Mifepristone is commonly utilized for recurrent CD, ESA with unidentified lesions or when surgery is not an option, and advanced adrenocortical cancer. It is effective in rapidly alleviating severe hypercortisolemia in patients with CS [14].

Mifepristone, approved by the US Food and Drug Administration (FDA) in 2012, acts as a competitive antagonist for the glucocorticoid receptor (GR) and progesterone receptor (PR). It is used to manage hyperglycemia in patients with endogenous hypercortisolism (CS) who also have type 2 diabetes mellitus or glucose intolerance and have either failed surgery or are not suitable candidates for it [15]. Recommendations are provided for handling various effects associated with mifepristone, including symptoms of cortisol withdrawal, hypokalemia, changes in thyroid function, antiprogesterone activity-related effects, and rash [16]. Close patient monitoring and clinical judgment are essential for the safe and effective use of mifepristone to ensure optimal clinical outcomes, particularly in elderly patients with multiple chronic diseases who are on multiple medications. Adverse reactions should be closely monitored during treatment.

This patient presents a complex case with multiple chronic diseases, geriatric syndromes, and a vacuolated sella turcica. Due to the high risks and uncertain benefits associated with a transnasosphenoidal approach for resecting the pituitary adenoma, a decision was made after thorough consideration of risks and benefits through shared decision-making between healthcare providers and the patient. The incidence of CS in the elderly is low [17], often presenting with atypical clinical manifestations and limited research data. In elderly patients presenting with multiple complications of CS, such as hypertension, diabetes, osteoporosis, and thromboembolic diseases, along with recent suspicious clinical manifestations such as thin skin, easy bruising, and changes in body fat distribution, the diagnosis of CS should be considered. This is particularly crucial if patients also exhibit hypokalemia, adrenal gland enlargement, or adrenal gland mass on auxiliary examinations. For elderly patients unsuitable for pituitary surgery, oral mifepristone, an antagonist glucocorticoid therapy, is a viable treatment option. Long-term monitoring is essential to assess the patient's disease progression and prognosis.

## Conclusions

This case report discusses a rare occurrence of CD in an elderly patient with multiple chronic conditions. The patient was initially admitted for severe respiratory symptoms and diagnosed with an acute exacerbation of chronic obstructive pulmonary disease. However, further physical examination post-admission revealed signs such as supraclavicular fat pad, buffalo back, and scattered ecchymoses on both upper limbs, prompting suspicion of CS. Subsequent endocrine tests confirmed a diagnosis of CD due to a pituitary microadenoma. A comprehensive geriatric assessment was conducted, considering the patient's functional status, comorbidities, treatment risks and benefits, and personal preferences. A tailored treatment plan was developed through shared decision-making. The importance of meticulous physical examination in detecting disease clues is highlighted, despite the availability of advanced imaging and laboratory tests. Long-term patient outcomes and prognosis require ongoing follow-up observations.
